# Comparative Expression Profiling and Sequence Characterization of ATP1A1 Gene Associated with Heat Tolerance in Tropically Adapted Cattle

**DOI:** 10.3390/ani11082368

**Published:** 2021-08-11

**Authors:** Muhammed Elayadeth-Meethal, Aravindakshan Thazhathu Veettil, Muhasin Asaf, Sathiamoorthy Pramod, Shane K. Maloney, Graeme B. Martin, M. Jordana Rivero, Veerasamy Sejian, Punnoth Poonkuzhi Naseef, Mohamed Saheer Kuruniyan, Michael R. F. Lee

**Affiliations:** 1 Department of Animal Breeding and Genetics, Kerala Veterinary and Animal Sciences University, Pookode, Wayanad 673576, Kerala, India; muhasin@kvasu.ac.in; 2 Livestock Research Station, Thiruvazhamkunnu, Palakkad 678601, Kerala, India; pramod.s@kvasu.ac.in; 3 UWA School of Agriculture and Environment, University of Western Australia, Crawley, WA 6009, Australia; graeme.martin@uwa.edu.au; 4 Centre for Advanced Studies in Animal Genetics and Breeding, Kerala Veterinary and Animal Sciences University, Pookode, Wayanad 680651, Kerala, India; aravindakshantv@kvasu.ac.in; 5 School of Human Sciences, University of Western Australia, Crawley, WA 6009, Australia; shane.maloney@uwa.edu.au; 6 Rothamsted Research, North Wyke, Devon EX20 2SB, UK; jordana.rivero-viera@rothamsted.ac.uk; 7 ICAR-National Institute of Animal Nutrition and Physiology, Adugodi 560030, Bangalore, India; drsejian@gmail.com; 8 Department of Pharmaceutics, Moulana College of Pharmacy, Perinthalmanna 679321, Kerala, India; drnaseefpp@gmail.com; 9 Department of Dental Technology, College of Applied Medical Sciences, King Khalid University, Abha 61421, Saudi Arabia; mkurunian@kku.edu.sa; 10 School of Sustainable Food and Farming, Harper Adams University, Edgmond, Newport TF10 8NB, UK; MRFLee@harper-adams.ac.uk

**Keywords:** climate resilience, livestock, expression profiling, molecular clock, ATP1A1 gene, body size, heat tolerance, marker assisted selection

## Abstract

**Simple Summary:**

An understanding of the way that animals respond to heat stress is key to the development of adaptation and mitigation strategies for a changing climate. The response of mammals to heat exposure involves changes at every level of organization from molecular and cellular to systemic and behavioral. The concert of events involves many genes and gene products. The Na+/K+ ATPase ∝1 (ATP1A1), a product of the *ATP1A1* gene, is important for the response to heat because it determines the activity of the Na^+^/K^+^ pump that is ubiquitous in cell membranes. It was shown recently that *ATP1A1* is important in combating the oxidative stress that a cell faces and that it modulates the Src signaling pathway that is involved in the response to many stressors. Vechur cattle (dwarf *Bos taurus indicus*) are well known for their adaptability to the tropical heat and humidity that persists in their native state of Kerala, India. We here analyze the comparative expression profile of the *ATP1A1* gene in heat-tolerant Vechur and Kasaragod (another dwarf *B. t. indicus*) cattle and a heat-intolerant crossbreed (*B. t. taurus* × *B. t. indicus*) and characterize the sequence of *ATP1A1* mRNA in the Vechur genotype. Environmental stress and heat tolerance were measured. Expression profiling indicated that *ATP1A1* was differentially expressed in the phenotypically disparate cattle breeds. A molecular evolutionary genetic analysis revealed that the divergent origin of dwarf cattle was adaptive in response to heat stress and suggests the potential use of *ATP1A1* as a marker for heat tolerance.

**Abstract:**

Climate change is an imminent threat to livestock production. One adaptation strategy is selection for heat tolerance. While it is established that the *ATP1A1* gene and its product play an important role in the response to many stressors, there has been no attempt to characterize the sequence or to perform expression profiling of the gene in production animals. We undertook a field experiment to compare the expression profiles of *ATP1A1* in heat-tolerant Vechur and Kasaragod cattle (*Bos taurus indicus*) with the profile of a heat-susceptible crossbreed (*B. t. taurus* × *B. t. indicus*). The cattle were exposed to heat stress while on pasture in the hot summer season. The environmental stress was quantified using the temperature humidity index (THI), while the heat tolerance of each breed was assessed using a heat tolerance coefficient (HTC). The *ATP1A1* mRNA of Vechur cattle was amplified from cDNA and sequenced. The HTC varied significantly between the breeds and with time-of-day (*p* < 0.01). The breed–time-of-day interaction was also significant (*p* < 0.01). The relative expression of *ATP1A1* differed between heat-tolerant and heat-susceptible breeds (*p* = 0.02). The expression of *ATP1A1* at 08:00, 10:00 and 12:00, and the breed–time-of-day interaction, were not significant. The nucleotide sequence of Vechur *ATP1A1* showed 99% homology with the *B. t. taurus* sequence. The protein sequence showed 98% homology with *B. t. taurus* cattle and with *B. grunniens* (yak) and 97.7% homology with *Ovis aries* (sheep). A molecular clock analysis revealed evidence of divergent adaptive evolution of the *ATP1A1* gene favoring climate resilience in Vechur cattle. These findings further our knowledge of the relationship between the *ATP1A1* gene and heat tolerance in phenotypically incongruent animals. We propose that *ATP1A1* could be used in marker assisted selection (MAS) for heat tolerance.

## 1. Introduction

Tolerance to high heat and humidity is a vital functional trait in species that evolved in adverse environments [1,2,3] and is mediated by molecular mechanisms that demonstrate the complex interplay of genes and environment [4,5]. In this context, the relationship between body size and tolerance to heat stress becomes critical [6] and, in southern India, is exemplified by the indigenous dwarf *B. t. indicus* cattle—the Vechur, the smallest cattle genotype in the world, and the slightly larger Kasaragod—that are mainly reared in the integrated farming systems that prevail in the region. Smaller size and tolerance to heat stress evolved in parallel as an adaptation to the tropical climate prevailing in their native habitat [6]. As a consequence, these genotypes are considered to be an important genetic resource for selection for climate resilience [7,8].

Heat stress results in plasma electrolyte imbalance that is caused mainly by oxidative stress, and the genes associated with stress response and protein repair are upregulated in stressed animals, whereas the genes associated with biosynthesis, metabolism, and body conformation are downregulated [9]. A key to the response is the heat shock proteins (HSP), the expression of which increases while the expression of non-HSPs decreases [10]. For these reasons, the differential expression of the genes regulating these processes is considered as a measure of cellular stress [11].

Of particular interest is the gene controlling Na^+^/K^+^ adenosine triphosphatase (Na^+^/K^+^ ATPase), a cell membrane protein that controls membrane permeability by coupling the transport of three Na^+^ ions outward and two K^+^ ions inward [12]. This coupling of ions across the plasma membrane is achieved using energy released from ATP hydrolysis [13,14,15]. The enzyme has three subunits, alpha, beta, and gamma, among which the alpha subunit is the largest and possesses the site for ATP hydrolysis. There are four alpha ATPases, *ATP1A1*, *ATP1A2*, *ATP1A3,* and *ATP1A4*, that code for α1, α2, α3, and α4 protein subunits, respectively. The expression of *ATP1A1* is ubiquitous and is important in maintaining Na^+^ and K^+^ homeostasis across the plasma membrane of all tissues in the body, including blood cells [16].

Cell membrane permeability is affected by heat stress because there are changes in the electrochemical gradient that are mediated, at least partly, by changes in the expression of the Na^+^/K^+^ ATPase [15]. The major isoform of this enzyme, α1, encoded by the *ATP1A1* gene, is a prominent non-heat shock protein that is associated with the response to heat stress [17]. Alternative splicing of *ATP1A1* mediates the HSP-mediated heat shock response by maintaining the ATP balance [18,19]. Thus, *ATP1A1* is considered a key non-HSP gene marker for tolerance to heat stress [16]. Indeed, the bovine *ATP1A1* gene, with a coding sequence of 3065 nucleotides scattered over 23 exons, has polymorphisms that are associated with heat tolerance traits in dairy cattle [20,21]. Polymorphisms in *ATP1A1* are associated with heat tolerance, disease susceptibility, and fertility in several breeds of dairy cattle, including Jersey crossbred, Sahiwal, Holstein, and Cholistani [22,23,24,25,26,27,28,29]. In beef cattle, heat stress is associated with changes in the expression of *ATP1A1* [30,31,32].

Glucocorticoids increase Na^+^/K^+^ ATPase mRNA levels in skeletal muscles [33]. Buckard et al. (2015) analyzed the role of *ATP1A1* in signal transduction involving cardiotonic binding induced signals through an Src signaling mediated pathway [34]. The cardiotonic steroids digoxin, ouabain, and bufarin act as specific inhibitors of *ATP1A1* [35,36,37].

Sequence characterization, molecular evolutionary analysis, and molecular clock analysis enable the identification of the selective pressures and evolutionary history of particular taxa in relation to their environment [3]. In the genus *Bos,* functional traits such as body size and tolerance to stress and diseases evolved in parallel in response to the environmental adversities in which the breeds evolved [6]. The molecular clock accounts for the genetic distance between the taxa and translates distance to the time using calibration rate, measured in terms of rate of genetic change per unit of time [3].

Thus, advances in genome sequencing, genome-wide association studies, and evolutionary analysis enabled the unravelling of the mechanistic ways by which animals gain tolerance to adverse climatic conditions. The knowledge at the molecular level of a key candidate gene (*ATP1A1*) in cellular metabolism advances this knowledge and helps to select animals with high tolerance. However, although the *ATP1A1* gene plays a key role in metabolism, growth, production, reproduction, and environmental adaptation of animals, the molecular genetics and the evolutionary basis of the role of *ATP1A1* in animals with different body size and heat tolerance are not clearly understood. Hence, this study was designed: (1) to assess heat tolerance in Vechur, Kasaragod and crossbred cattle using a heat tolerance coefficient (HTC); (2) to compare the differential expression profile of *ATP1A1* in heat stressed animals; (3) to sequence, characterize, and compare the *ATP1A1* mRNA in Vechur cattle; and (4) to perform molecular evolutionary analysis to study the evolution of the *ATP1A1* gene.

## 2. Materials and Methods

Ten adult female animals each of Vechur, Kasaragod, and crossbred cattle were selected for the study. The Vechur and the Kasaragod animals were obtained from the Kerala Veterinary and Animal Sciences University Vechur Conservation Unit, and the crossbred animals were obtained from the Kerala Veterinary and Animal Sciences University livestock farm, Mannuthy. All animals were previously exposed to heat challenge conditions because they grazed outside in summer. During the experiment, the animals were allowed to graze on hot summer days without any shade and were given a regular diet of concentrate feed, with drinking water *ad libitum*. The study was approved by the institutional animal ethics committee of the Kerala Veterinary and Animal Sciences University. The detailed experimental design is explained in a previous article [6].

Temperature and relative humidity data were obtained from an automatic weather station at the Kerala Agricultural University about four kilometers from the location of the experiment. The environmental stress was evaluated using the temperature humidity index (THI): THI = (1.8 × T_a_ + 32) − (0.55 − 0.0055 × RH) × (1.8 × T_a_ − 26), where T_a_ = atmospheric temperature (°C) and RH = percent relative humidity [38]. Respiratory rates (RR) and rectal temperatures (RT) were recorded at half hour intervals from 08:00 to 14:00 for ten days. The heat tolerance of the animals was assessed using a heat tolerance coefficient (HTC): HTC = RR/23 + RT/38 [16].

Jugular blood (5 mL) was sampled from each animal three times each day at 08:00, 10:00, and 12:00 using a vacutainer containing 5 mg EDTA as anticoagulant under aseptic conditions. The samples were immediately placed on ice and transported to the laboratory where RNA was extracted using GeneiPure RNA kits (Cat. No. KT-173, Genei, Bangalore, India). The quality of the RNA was checked using gel electrophoresis, and RNA was quantified using a spectrophotometer (Nanodrop ND-1000, Thermo-Scientific, Waltham, MA, USA). A DNase1 kit (Cat. No. AMP-D1, Sigma-Aldrich, St. Louis, MO, USA) was used to inhibit DNAse, followed by cDNA synthesis using kits (Cat. No. K1621, Fermentas, Waltham, MA, USA).

### 2.1. Quantitative Real Time PCR (q-RT PCR)

The q-RT PCR experiment was designed to test whether gene expression differed between Vechur, Kasaragod, and crossbred animals at various levels of heat stress (Table 1). Extended blood sample storage or delays in processing due to technical issues reduced *ATP1A1* expression, indicating a need for fresh blood samples and speedy processing for optimal measures of ATP1A1 expression. A nested design (hierarchal or clustered) was followed in the present study, which consisted of three animals from each group, from which three treatment samples were collected and extracted. After the RT reaction, each sample was split into three q-RT PCR reactions for effective distribution of variance components [39]. To compare gene expression profiles under thermal stress, the selection of appropriate reference genes was important [40]. In our study, ß-actin was used as the reference gene, as it was stably expressed during thermal stress compared to other usual housekeeping genes GAPDH and 18S rRNA [40,41,42].

The q-RT-PCR used custom synthesized primer pairs using the mRNA sequence NM_1076798.1- F- TCCTCATCGGCATCATTGTAGCCA; R- AGCCTCCAGGTTCTTCACTAAGCA to amplify a 122 bp amplicon with an annealing temperature of 60 °C. Gene expression was profiled using Illumina Eco^®^ q-RT PCR with SYBR green. The profiling relied on the comparison of expression of ATP1A1 with expression of the reference gene (ß-actin) to calculate ΔCt; the expression of the same gene in treatment samples (10:00 and 12:00) was compared with expression in control (calibrator) samples (at 08:00) to calculate ΔΔCt. For calculation of the relative expression, the comparative C_t_ method, also known as the 2^−∆∆Ct^ method, was used: ∆∆C_t_ = ∆C_t,sample_ − ∆C_t,calibrator_ (∆C_t_ = Ct value for the target gene − Ct value of the reference gene). The fold changes in expression of ATP1A1 gene after exposure to heat stress were estimated by comparison with values of the control animals (before exposure) using the above formula RQ = 2^−∆∆Ct^. For q-RT-PCR, we used Maxima SYBR Green master mix (Cat. No. K0221, Thermo Scientific).

Initially, the PCR conditions were optimized by using various concentrations of MgCl_2_ and dNTPs and by testing various time–temperature combinations for annealing and extension. The combination that gave the best result in terms of yield and specificity of the product was further used to amplify the samples for q-RT PCR and PCR in a thermal cycler (Bio-Rad, Thermal Cycler^TM^, Hercules, CA, USA). The PCR products were confirmed by gel electrophoresis using ethidium bromide staining in 2% agarose gel documented in a gel documentation system (Bio-Rad, Hercules, CA, USA).

For q-RT-PCR, separate PCR reactions were set up for *ATP1A1* and ß-actin genes. First, a master mix was prepared for the required number of reactions. The Illumina Eco^®^ q-RT PCR system utilized 48-well micro-plates for the reactions. The template (cDNA) was loaded separately into designated wells. Initial denaturation at 95 °C for 10 min was followed by denaturation at 95 °C for 15 s and repeated for 35 cycles. Data recording was done at the annealing stage set 60 °C for 60 s. The reaction specificity was assessed using melt curve analysis that consisted of denaturation at 95 °C for 15 s and annealing at 55 °C for 15 s followed by 95 °C for 15 s. The melt curve data were recorded in the denaturation step. The data were analyzed for relative quantification using the ΔΔCt method.

### 2.2. Amplification of the ATP1A1 Gene

The *ATP1A1* mRNA of Vechur cattle was amplified from cDNA and sequenced. Primers were designed to amplify the ATP1A1 gene based on the mRNA sequence NM_1076798.1 using the primer blast tool at NCBI. The primers were checked for integrity using Sequence Manipulation Suite software (http://www.bioinformatics.org/sms2/html). The two primers selected (F-TATGGGGAAGGGGGTTGGACGTGATA; R-GTCGTTTCCACAGACGGATGTTTCTC) were custom-synthesized by Sigma-Aldrich.

The PCR conditions were optimized by using various concentrations of MgCl_2_ and dNTPs and by testing various time–temperature combinations for annealing and extension. JumpStart^®^ *Taq* DNA polymerase (Cat. No. D-9307, Sigma-Aldrich) was used for amplification. The PCR was carried out in a volume of 50 µL in a 200 µL PCR tube. The combination that gave the best result in terms of yield and specificity of the product was further used for amplification of the samples, and PCR was carried out in a thermal cycler (Bio-Rad, Thermal Cycler^TM^, Hercules, CA, USA). The PCR conditions were as follows: initial denaturation at 96 °C for 30 s, followed by 35 cycles with denaturation at 94 °C for 15 s; primer-specific annealing temperature of 60 °C 30 s to specifically amplify the target region; extension at 68 °C for 3.5 min followed by final extension at 68 °C for 25 min. The PCR product was checked with 0.8% agarose gel prepared in 1X TAE buffer and incorporated with ethidium bromide along with a 1 Kb ladder (GeneRuler^TM^ Cat. No. SM0311, ThermoFisher Scientific, Waltham, MA, USA). The gels were electrophoresed in 1X TAE buffer for 45 min at 75 V (5 V/cm) and documented in a gel documentation system (Bio-Rad, Hercules, CA, USA).

### 2.3. Sequence, Molecular Clock, and Data Analysis

The PCR-amplified *ATP1A1* gene from Vechur cattle was sequenced and assembled commercially by Bioserve Biotechnologies (India) Pvt. Ltd. (Hyderabad, India) using the dideoxynucleotide chain termination sequencing method in an automated DNA sequencer (Applied Biosystems, ThermoFisher Scientific, Waltham, MA, USA). The raw sequence data were verified using the chromatogram to obtain clean sequence data. For comparison of similarity between the sequences, multiple sequence alignment feature of Clustal W2 was used to obtain the alignments, and a paired BLAST search was carried out for each pair to confirm paired sequence similarities. BLASTn analysis was done to obtain sequence similarity with nucleotide sequences available on Genbank. Most of these programs were in the CoreSuit 10 software DNASTAR Inc. (Madison, WI, USA), available at the bioinformatics laboratory of Department of Animal Breeding, Genetics and Biostatistics, College of Veterinary and Animal Sciences, Mannuthy, India.

The 3287 bp sequence obtained was analyzed for homology by BLAST search at the National Centre for Biotechnology Information (NCBI) site using BLAST program (http://www.ncbi.nlm.nih.gov/BLAST). The raw *ATP1A1* sequences were annotated based on the bovine *ATP1A1* (Accession # NM_ 001076798.1) gene. The Sequence Manipulation Suite was used for generating, formatting, and analyzing DNA and protein sequences. This program is available at http://www.bioinformatics.org/sms2/html. Multiple sequence alignment was done using the European Bioinformatics Institute (EBI) tool, Clustal (http://align.genome.jp/), and the SPLIGN program available at the NCBI site [43]. Using SPLIGN, the sequence was compared simultaneously with the sequence in the *Bos t. taurus* reference genome (1378962611).

For molecular clock analysis, the evolutionary history of the *ATP1A1* gene was inferred using the neighbor-joining method [44]. The optimal tree with the sum of branch length = 0.12680380 was selected. The percentage of replicate trees in which the associated taxa clustered together in the bootstrap test (1000 replicates) was shown next to the branches [45]. The evolutionary distances were computed using the maximum composite likelihood method and are presented in the units of the number of base substitutions per site [46]. This analysis involved 9 nucleotide sequences. Codon positions included were 1st+2nd+3rd+noncoding. All ambiguous positions were removed for each sequence pair (pairwise deletion option). There was a total of 3871 positions in the final dataset. Evolutionary analyses were conducted in MEGA X [47,48]. 

Subsequently, a timetree was inferred by applying the RelTime method in the constructed phylogenetic tree [49,50]. The branch lengths were calculated using the maximum likelihood (ML) method and the Tamura-Nei substitution model [51]. The timetree was computed using 1 calibration constraint—the split of *B. t. taurus* and *B. t. indicus*. The method of Tao et al. (2019) was used to set minimum and maximum time boundaries on nodes for which calibration densities were provided [52]. The estimated log likelihood value of the tree was −8754.60. The human *ATP1A1* nucleotide sequence was taken as an outgroup. Codon positions included were 1st+2nd+3rd+noncoding. 

The statistical analysis was done in R (Version 4.1.0) [53]. Values for HTC and relative expression were log-transformed before analysis of variance. The effects of breed, period, and period–breed interaction was estimated. Estimated marginal means are presented. The effect of THI on HTC was also compared among animal groups. Pair-wise comparison was done using the TukeyHSD test in R. The level of significance was set at *p* < 0.05.

## 3. Results

**Figure 1:**
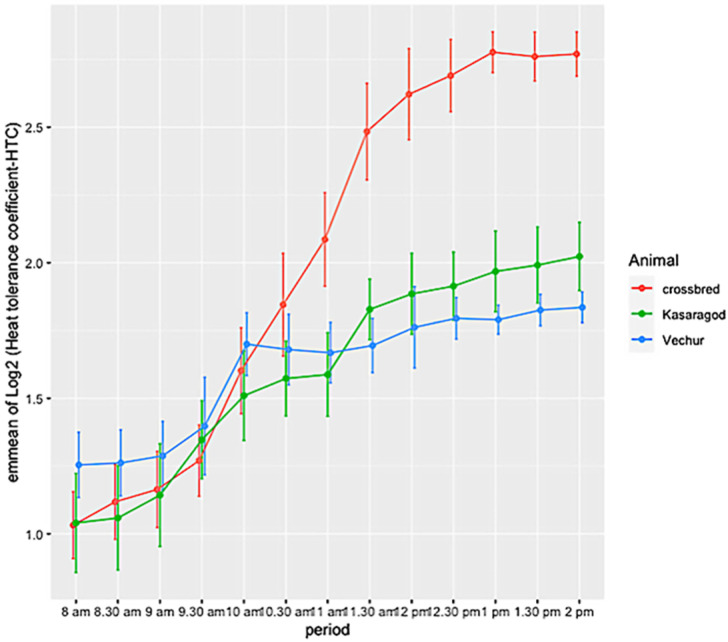
Estimated marginal means (emmean) of the heat tolerance coefficient (HTC) in crossbred, Kasaragod, and Vechur cattle, from 08:00 to 14:00.

**Figure 2:**
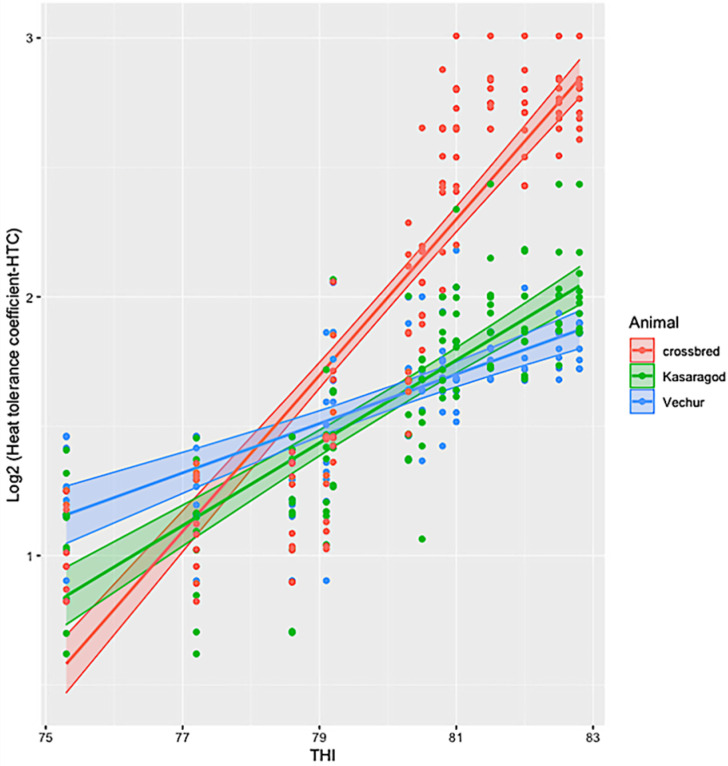
Estimated marginal means of heat tolerance coefficient (HTC) in crossbred, Kasaragod, and Vechur cattle at different THI levels.

**Figure 3:**
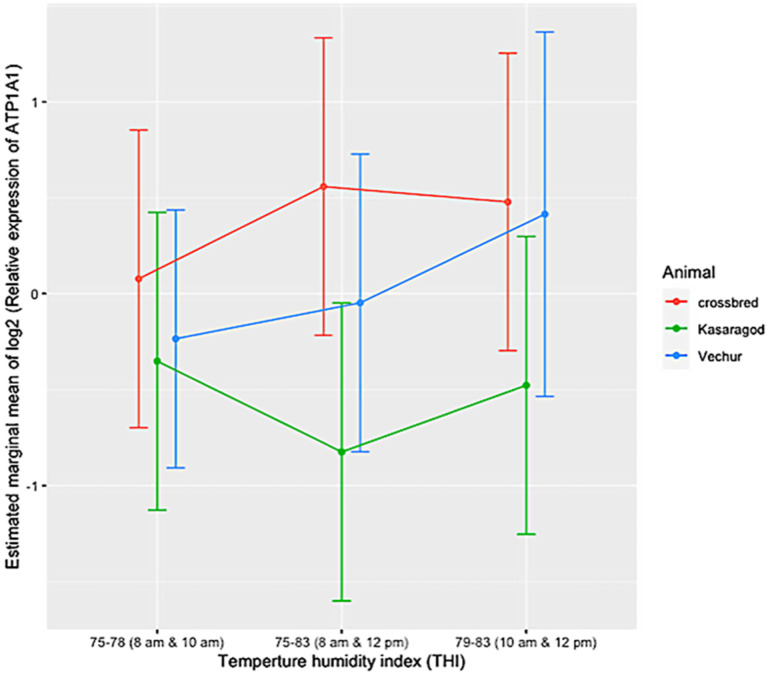
Relative expression of the *ATP1A1* gene in Vechur, Kasaragod, and crossbred cattle during different periods from 08:00 h to 12:00 h. Period 1 was between 08:00 and 10:00; period 2 was between 10:00 and 12:00; period 3 was between 08:00 and 12:00. The estimated marginal mean is shown on the y axis. The expression profile of ATP1A1 varied significantly among the breeds (p = 0.02). However, the effects of time and the time x breed interaction were not significant. Pair-wise comparison of ATP1A1 expression levels in different lineages showed significant difference in expression between Kasargode and crossbred cattle (*p* = 0.009).

The ambient temperature and humidity increased progressively from 08:00 to 14:00. The maximum air temperature during the study period was 31.1 ° C, the relative humidity was 83.8%, the wind speed was 5.8 m/s, and the solar radiation was 1.3 MJ. The calculated THI increased from 75 at 08:00 to 83 at 14:00. There was significant variation in HTC between the groups (*p* < 0.01). The effects of period and period–breed interaction was also significant (*p* < 0.01) (Figure 1 and Figure 2). Compared to dwarf cattle, the HTC increased in crossbred animals when the THI increased (Figure 1 and Figure 2). The estimated marginal means (emm) of *ATP1A1* relative expression are given in Table 2. The expression profile of *ATP1A1* varied significantly between the breeds (*p* = 0.02). However, the period and the period–breed interactions were not significant for *ATP1A1* expression (Figure 3). Pair-wise comparison of *ATP1A1* expression levels in different lineages showed significant difference in expression between Kasargode and crossbred cattle (*p* = 0.009).

**Figure 4:**
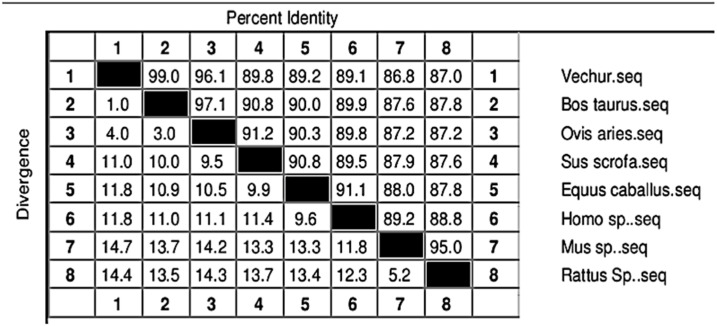
Percent identity (upper half) and divergence (lower half) of the ATP1A1 gene of Vechur cattle with similar sequences in the database.

**Figure 5:**
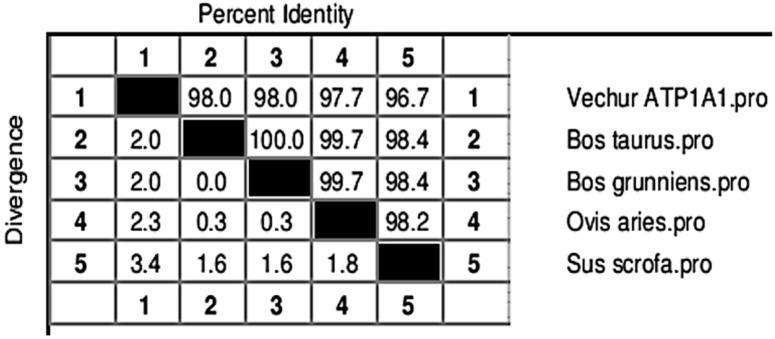
Percent identity (upper half) and divergence (lower half) of the predicted ATP1A1 protein sequence of Vechur cattle with similar sequences in the database.

**Figure 6:**
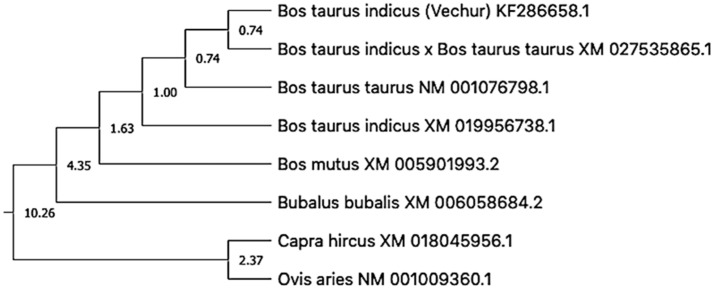
Timetree depicting the evolutionary molecular clock of the *ATP1A1* gene in dwarf Vechur (*Bos taurus indicus*) cattle. The branch lengths were calculated using the maximum likelihood (ML) method and the Tamura-Nei substitution model inferred by applying the RelTime method.

The gel image of 3287 bp PCR amplified *ATP1A1* gene is given in Figure S1. The sequence is available in NCBI (Accession number KF286658.1). Details of genomic structure and coordinates of *ATP1A1* in Vechur are given in Table 3. A total of 36 variations in the *ATP1A1* gene of Vechur were detected when compared with *Bos t. taurus* (Figures S2–S4). The percent identity and the divergence of the *ATP1A1* gene and protein are given in Figure 4 and Figure 5. The protein sequence showed 98% homology with *B. t. taurus* cattle and *B. grunniens* (yak) and 97.7% with *Ovis aries* (sheep). Pair-wise genetic distances between the taxons are given in Table 4. The calculated genetic distances between Vechur, *B.t. taurus,* and *B.t. taurus* × *B. t. indicus* were 0.0076 and 0.0081, respectively. The evolutionary history of the *ATP1A1* gene in Vechur cattle, inferred using the neighbor-joining method, is given in Figure S5. The optimal tree with the sum of branch length = 0.12680380 is shown. The percentage of replicate trees in which the associated taxa clustered together in the bootstrap test (1000 replicates) is shown next to the branches. The timetree depicting the evolutionary molecular clock of the *ATP1A1* gene in dwarf Vechur (*B. t. indicus*) cattle is given in Figure 6. The phylogenetic tree shown in Figure S5 was used for molecular clock analysis. The branch lengths were calculated using the maximum likelihood (ML) method and the Tamura-Nei substitution model inferred by applying the RelTime method. Molecular clock analysis revealed the ancient origin of Vechur cattle compared to *Bos t. taurus* (Figure 6).

## 4. Discussion

Under thermoneutral conditions, all three groups of animals showed similar physiological responses, as indicated by the HTC. However, as the heat load increased, the HTC became higher in the crossbred cattle than in the dwarf Vechur and Kasaragod animals. Using expression profiling, we found that the expression of the *ATP1A1* gene significantly varied between the heat-tolerant dwarf Vechur and Kasaragod cattle and the heat stress susceptible, standard sized, crossbred cattle. However, when THI increased, among the dwarf cattle lineages (Vechur and Kasaragod), Vechur had a higher level of *ATP1A1* expression compared to Kasaragod animals. Although superior thermotolerance of dwarf cattle was evident in their physiological responses, the differential *ATP1A1* expression upon increasing heat load among dwarf lineages requires further investigation.

The molecular clock analysis revealed separate clustering of Vechur cattle along with Bison and *B. taurus* cattle. Previous molecular clock analysis revealed that taurine and indicine cattle diverged around 1 mya [54,55]. The rate of DNA evolution and, hence, the molecular clock are primarily affected by body size and temperature [56,57,58]. The smaller body size of Vechur cattle might favor faster adaptive evolution to high heat and humidity in their lineage [59].

The experiment was conducted from 08:00 to 14:00, as the physiological responses were found to be maximum at 14:00 and minimum at 02:00 during all seasons [60]. The THI values reached a maximum in the afternoon, and the minimum was early in the morning [61]. Cattle are generally considered stressed above a THI of 72 [62]. The THI is similar to UTCI (universal thermal climate index) that is used in humans [63]. It can be used to account for the effect of heat stress on production of dairy cattle [64]. The THI is used as an indicator of milk production losses due to heat stress, and indices with larger weights on humidity are more suitable for humid climates [65]. The THI threshold refers to a value at which detectable change begins in the animal, denoted by THI_on_. The THI does not include important climatic variables such as solar radiation and wind speed. Likewise, it does not include management factors (the effect of shade) or animal factors (genotype differences).

Climate resilience is related to metabolic efficiency and body size of the organism, as physiological and genomic properties scale with body size [66]. Species that are less metabolically efficient have a larger metabolic heat load to deal with, which becomes difficult when the environment is not conducive to heat loss by dry or evaporative routes, such as hot and humid conditions exemplified by THI higher than 72 [67]. More metabolically efficient species/breeds have the potential to tolerate acute and chronic climate stress [68,69,70]. Heat shock proteins (HSPs) are studied in detail in relation to the enhanced thermotolerance [71]. Due to low specificity of HSPs, other non-HSPs were suggested as markers of thermotolerance. Hence, non-heat shock proteins such as *ATP1A1* are considered candidate genes for thermotolerance and suggested as markers for selection for heat tolerance, disease resistance, low carbon foot print, conservation, and sustainable intensification [10,16]. To assess the impact of climate change on animals, several genetic markers were suggested [10]. Na^+^/K^+^ ATPase is the major class of enzymes that are involved in the membrane permeability of cells. An increased expression of *ATP1A1* mRNA during prolonged exercise was reported without a corresponding increase in protein levels [72]. *ATP1A1* is extensively studied in exercise physiology and inactivity in humans [73]. *ATP1A1* interacts with other proteins and modulates hypertension and feed intake [74]. 

During thermal stress, multiple gene networks are activated [75]. Thus, the changes in *ATP1A1* expression that we observed in the present study may have been influenced by many factors such as increased expression of heat shock factors and heat shock proteins, altered metabolism of glucose, amino acids, and fat, and altered immune responses [10]. Previous studies revealed breed specific nucleotide diversity and expression patterns of *ATP1A1* in Indian goats and buffaloes [76,77]. Hence, *ATP1A1* could be used as a marker for selection for heat tolerance [70].

Molecular clock theory provides a way to estimate variation in the rate of molecular evolution [78,79]. Several methods can be used to calibrate the molecular clock, including complex geological events [80]. In the case of vertebrates, body size and temperature significantly affect the molecular clock and thus in combination influence metabolism and the rate of evolution [56,57,58]. Species with a smaller dispersal range and large body mass tend to experience niche shrink in response to environmental perturbations and may require adaptive or plastic tolerance to survive in a warming climate [81]. The relative synonymous substitution rate after adjusting for taxa-specific and G+C constituent influences is fast for *ATP1A1* [82]. Vechur, being the smallest breed of cattle, compared with standard size crossbred cattle make an ideal model for the evolutionary molecular clock analysis of large ruminants.

The sequence data revealed differences in the mRNA sequence of the *ATP1A1* gene in Vechur cattle compared to *B. t. taurus* cattle, which was possibly one of the reasons for altered expression of these genes in Vechur cattle during thermoneutral condition. The 5′ and the 3′ UTRs of eukaryotic mRNAs were shown to influence several aspects of gene expression [83]. The 3′ UTR influences mRNA stability and translation initiation and, hence, mutations in the 3′ UTR are associated with a number of diseases [84].

Selection for heat stress is possible and particularly effective for environments with a high average THI. Continued selection for production ignoring heat tolerance results in a decrease in heat tolerance, as the genetic correlation between general production and heat tolerance is around −0.3. Thus, a combined selection for production and heat tolerance is possible [64]. For crossbreeds, the ability to acclimate is limited. The findings of the current study suggest that crossbreeds maximized their molecular safety factors, as inferred from the higher levels of expression of ATP1A1 at thermoneutral conditions compared to dwarf cattle, which does not allow for further adjustments to current changes in climate [85,86,87,88]. These animals are living close to their stress tolerance limit, and a global warming scenario is likely to push them beyond that which may ultimately lead to further decrease in productivity [89,90].

Thus, the first time, we have provided a quantitative fingerprint of expression of *ATP1A1* genes in thermotolerant Vechur and Kasaragod cattle compared to thermally sensitive crossbred cattle under thermoneutral and heat stress conditions. The method is reproducible and sensitive enough to detect different levels of expression of *ATP1A1* in different thermal conditions. The results have implications for cattle in a global warming scenario, as alleles for thermotolerance can be detected by allele mining or expression QTL (eQTL) to develop thermotolerant breeds in future [89,90].

## 5. Conclusions

The performance of livestock is evaluated in three key areas: growth, production, and reproduction. To maintain thermal equilibrium, cattle rely on a delicate balance of heat production and heat loss. Mechanisms of adaptation include morphological, behavioral, biochemical, and physiological changes, and the capacity of animals to adapt is limited primarily by their genetic makeup. The stress response is influenced by a number of factors including species, breed, age, sex, previous exposure to stress, health status, performance, and body condition. Subsequent acclimatization may alleviate the strain that develops in response to stress, but performance may not return to pre-stress levels. In the present study, it was found that *ATP1A1* expression was significantly associated with heat tolerance in different populations studied. Additionally, we emphasize the potential use of *ATP1A1* as a marker for marker assisted selection (MAS) for heat tolerance.

## Figures and Tables

**Table 1:** Distribution of variance components followed in the gene expression study [39].

Confounding Variance			Studied Variance
Components	Inter Subject Variance	Processing Noise	Treatment Effect
Source	1. Different base line expression 2. Different response to treatment	1. Sampling 2. RT 3. qPCR	Difference between groups induced by treatment
Intervention	1. Randomised 2. Used appropriate sample size 3. Used paired measures	1. Used replicates 2. Normalized to reference gene or spike	Maximized effect (by selecting heat tolerant Vechur and Kasaragod and heat susceptible crossbred cattle

**Table 2:** Estimated marginal means (EMM) of the relative expression of *ATP1A1* gene in different breeds of cattle at different periods from 08:00 to 12:00 (standard error = 0.37; df = 18). Period 1 was between 08:00 and 10:00; period 2 was between 10:00 and 12:00; period 3 was between 08:00 and 12:00.

Period	Breed	EMM	Lower CL	Upper CL
1	Vechur	−0.314	−1.092	0.4641
2	Vechur	0.2764	−0.502	1.0545
3	Vechur	−0.0479	−0.826	0.7302
1	Kasaragod	−0.352	−1.13	0.4261
2	Kasaragod	−0.4776	−1.256	0.3005
3	Kasaragod	−0.8246	−1.603	−0.0465
1	Crossbred	0.0773	−0.701	0.8554
2	Crossbred	0.4785	−0.3	1.2566
3	Crossbred	0.5586	−0.22	1.3367

**Table 3:** Details of genomic structure and coordinates of *ATP1A1*.

Exon	Genomic Coordinates	mRNA Coordinates	Length	Identity
Exon 1	918,643–918,655	1–13	13	100%
Exon 2	907,369–907,473	14–118	105	100%
Exon 3	906,689–906,748	119–178	60	100%
Exon 4	904,407–904,610	179–182	204	99.5%
Exon 5	903,621–903,734	383–496	114	100%
Exon 6	903,106–903,240	497–631	135	99.3%
Exon 7	902,879–902,996	632–749	118	97.5%
Exon 8	902,159–902,427	750–1018	269	98.5%
Exon 9	901,429–901,627	1019–1217	199	97%
Exon 10	901,068–901,177	1218–1327	110	99%
Exon 11	899,006–899,140	1328–1462	135	100%
Exon 12	898,152–898,344	1463–1655	193	99%
Exon 13	895,764–895,939	1656–1831	176	100%
Exon 14	894,642–894,778	1832–1968	137	100%
Exon 15	893,243–893,393	1969–2119	151	98.7%
Exon 16	892,478–892,648	2120–2288	169	100%
Exon 17	892,209–892,363	2289–2443	155	99.4%
Exon 18	891,714–891,837	2444–2567	124	98.4%
Exon 19	890,422–890,567	2568–2713	146	98.6%
Exon 20	890,166–890,296	2714–2844	131	100%
Exon 21	889,575–889,676	2845–2966	102	97.1%
Exon 22	887,420–887,511	2967–3038	92	98.9%
Exon 23	886,685–886,933	3039–3287	249	97.2%

**Table 4:** Pair-wise genetic distance between different lineages based on the mRNA sequence comparison of the *ATP1A1* gene.

Animal 1	Animal 2	Genetic Distance
KF286658.1 *Bos t. indicus* (Vechur)	NM 001076798.1 *Bos t. taurus*	0.0076
KF286658.1 *Bos t. indicus* (Vechur)	XM 027535865.1 *Bos t. indicus* × *Bos t. taurus*	0.0081
NM 001076798.1 *Bos t. taurus*	XM 027535865.1 *Bos t. indicus* × *Bos t. taurus*	0.0004
KF286658.1 *Bos t. indicus* (Vechur)	XM 005901993.2 *Bos mutus*	0.0094
NM 001076798.1 *Bos t. taurus*	XM 005901993.2 *Bos mutus*	0.0024
XM 027535865.1 *Bos t. indicus × Bos t. taurus*	XM 005901993.2 *Bos mutus*	0.0024
KF286658.1 *Bos t. indicus* (Vechur)	XM 010830730.1 *Bison bison*	0.0093
NM 001076798.1 *Bos t. taurus*	XM 010830730.1 *Bison bison*	0.0239
XM 027535865.1 *Bos t. indicus × Bos t. taurus*	XM 010830730.1 *Bison bison*	0.0252
KF286658.1 *Bos t. indicus* (Vechur)	XM 006058684.2 *Bubalus bubalis*	0.0163
NM 001076798.1 *Bos t. taurus*	XM 006058684.2 *Bubalus bubalis*	0.0084
XM 027535865.1 *Bos t. indicus × Bos taurus*	XM 006058684.2 *Bubalus bubalis*	0.0082
KF286658.1 *Bos t. indicus* (Vechur)	XM 018045956.1 *Capra hircus*	0.0287
NM 001076798.1 *Bos t. taurus*	XM 018045956.1 *Capra hircus*	0.0208
XM 027535865.1 *Bos t. indicus × Bos t. taurus*	XM 018045956.1 *Capra hircus*	0.0211
KF286658.1 *Bos t. indicus* (Vechur)	NM 001009360.1 *Ovis aries*	0.0299
NM 001076798.1 *Bos t. taurus*	NM 001009360.1 *Ovis aries*	0.0210
XM 027535865.1 *Bos t. indicus × Bos t. taurus*	NM 001009360.1 *Ovis aries*	0.0214
KF286658.1 *Bos t. indicus* (Vechur)	BC003077.2 *Homo sapiens*	0.0877
NM 001076798.1 *Bos t. taurus*	BC003077.2 *Homo sapiens*	0.0832
XM 027535865.1 *Bos t. indicus × Bos t. taurus*	BC003077.2 *Homo sapiens*	0.0835

## Data Availability

ATP1A1 mRNA sequence of Vechur cattle is available at NCBI (accession number KF286658.1).

## Supplementary Materials

The following are available online at https://www.mdpi.com/article/10.3390/ani11082368/s1, Additional materials and methods, Figure S1: Gel demonstrating the 3287 bp PCR amplified product of ATP1A1 gene of Vechur cattle in 1% agarose gel, Figure S2: Results of mRNA (cDNA) to genomic alignment created by Splign. Graphical view the alignment between Vechur ATP1A1 sequence KF 286658.1 and Bos taurus reference genome (1378962611). Figure S3: Tabular format for the alignment between Vechur ATP1A1 sequence KF 286658.1 and Bos taurus reference genome (1378962611). Figure S4: Segments and boundaries of the aligned regions of 23 exons between Vechur ATP1A1 sequence (KF 286658.1) and Bos taurus reference genome (1378962611). Figure S5: The evolutionary history of ATP1A1 gene in Vechur cattle inferred using the neighbor-joining method. The optimal tree with the sum of branch length = 0.12680380 is shown. The percentage of replicate trees in which the associated taxa clustered together in the bootstrap test (1000 replicates) are shown next to the branches.
