# Supplementary material for: Comparative Expression Profiling and Sequence Characterization of ATP1A1 Gene Associated with Heat Tolerance in Tropically Adapted Cattle
Source: Animals (Basel). 2021 Aug 11;11(8):2368. doi: 10.3390/ani11082368 (PMC8388727; doi:10.3390/ani11082368)
Supplement: Supplementary file 1 [file animals-11-02368-s001.zip › animals-1332528-supplementary.pdf]

Supplementary materials

Comparative Expression Profiling and Sequence Characterisation of ATP1A1 Gene Associated to Heat Tolerance in Tropically Adapted Cattle

Muhammed Elayadeth-Meethal[1, 3, 5*], Aravindakshan Thazhathu Veettil[2], Muhasin Asaf[1], S. Pramod[3], Shane K Maloney[4], Graem B Martin[5], M. Jordana Rivero[6], Veerasamy Sejian[7], Punnoth Poonkuzhi Naseef[3], Mohamed Saheer Kuruniyan[9], Michael R.F. Lee[10]

[1] Department of Animal Breeding and Genetics, Kerala Veterinary and Animal Sciences University, Pookode, Wayanad, Kerala, 673576, India. muhammed@kvasu.ac.in;
[2] Centre for advanced studies in Animal Genetics and Breeding, Kerala Veterinary and Animal Sciences University, Pookode, Wayanad, Kerala, 680651, India. aravindakshantv@kvasu.ac.in
[3] Livestock Research Station, Thiruvazhamkunnu, Palakkad, Kerala, 678601, India.pramod.s@kvasu.ac.in
[4]School of Human Sciences, University of Western Australia, Crawley, WA, 6009, Australia shane.maloney@uwa.edu.au
[5] UWA School of Agriculture and Environment, University of Western Australia, Crawley, WA, 6009, Australia graeme.martin@uwa.edu.au
[6] Rothamsted Research, North Wyke, Devon, UK EX20 2SB. jordana.rivero-viera@rothamsted.ac.uk
[7] ICAR-National Institute of Animal Nutrition and Physiology, Adugodi, Bangalore, 560030, India drsejian@gmail.com
[8] Department of Pharmaceutics, Moulana College of Pharmacy, Perinthalmanna, Kerala, 679321, India. drnaseefpp@gmail.com
[9] Dept. of Dental Technology. COAMS. King Khalid University. Abha, 61421, Saudi Arabia.mkurunian@kku.edu.sa
[10] Harper Adams University, Edgmond, Newport, TF10 8NB, UK. MRFLee@harper-adams.ac.uk
* Correspondence: muhammed@kvasu.ac.in

Table of contents

1. Additional materials and methods -q-RT-PCR
2. Figures S1-S5

1. Additional materials and methods -q-RT-PCR

For loading samples, the plate was placed into the Eco sample-loading dock, the notch was aligned with the matching indentation on the adapter. The dock light was turned on and the dock was inclined to a comfortable angle for pipetting. The samples and q-RT PCR reagents were pipetted into the plate and the plate was sealed with an Eco optical seal, which held the plate in place on the Eco sample loading dock, before the squeegee was dragged firmly across the surface to ensure the seal was secure. The plate adapter with sealed plate was placed into a plate centrifuge along with a second plate adapter for balance. The plate was centrifuged at 250 x g for 30 sec. The Eco lid was opened and the plate was placed on the block, and the notch aligned against the top left corner. The lid was closed and the reaction was started.

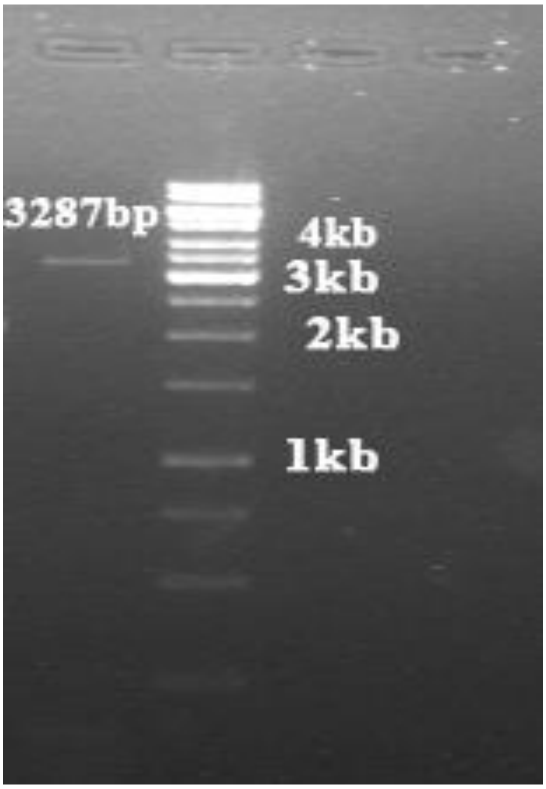

Figure S1. Gel demonstrating the 3287 bp PCR amplified product of ATP1A1 gene of Vechur cattle in 1% agarose gel (M- 1 Kb DNA Marker, A-ATP1A1 gene-3287bp)

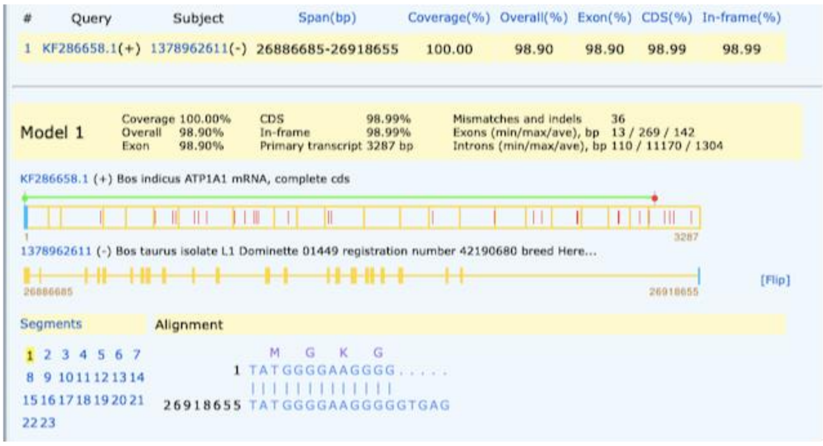

Figure S2. Results of mRNA (cDNA) to genomic alignment created by Splign. Graphical view the alignment between Vechur ATP1A1 sequence KF 286658.1 and Bos taurus reference genome (1378962611).

#	Query	Subject	Span(bp)	Coverage(%)	Overall(%)	Exon(%)	CDS(%)	In-frame(%)
1	KF286658.1(+)	1378962611(-)	26886685-26918655	100.00	98.90	98.90	98.99	98.99

#	Query	Subject	Idty	Len	Q.Start	Q.Fin	S.Start	S.Fin	Type	Details
+1	KF286658.1	1378962611	1	13	1	13	26918655	26918643	GC<exon>GT	M13
+1	KF286658.1	1378962611	1	105	14	118	26907473	26907369	AG<exon>GT	M105
+1	KF286658.1	1378962611	1	60	119	178	26906748	26906689	AG<exon>GT	M60
+1	KF286658.1	1378962611	0.995	204	179	382	26904610	26904407	AG<exon>GT	M194R[illegible]
+1	KF286658.1	1378962611	1	114	383	496	26903734	26903621	AG<exon>GT	M114
+1	KF286658.1	1378962611	0.993	135	497	631	26903240	26903106	AG<exon>GT	M134R
+1	KF286658.1	1378962611	0.975	118	632	749	26902996	26902879	AG<exon>GT	M8RM8[illegible]
+1	KF286658.1	1378962611	0.985	269	750	1018	26902427	26902159	AG<exon>GT	M82R2[illegible]
+1	KF286658.1	1378962611	0.97	199	1019	1217	26901627	26901429	AG<exon>GT	M8RM4[illegible]
+1	KF286658.1	1378962611	0.991	110	1218	1327	26901177	26901068	AG<exon>GT	M69RM[illegible]
+1	KF286658.1	1378962611	1	135	1328	1462	26899140	26899006	AG<exon>GT	M135
+1	KF286658.1	1378962611	0.99	193	1463	1655	26898344	26898152	AG<exon>GT	M18RM[illegible]
+1	KF286658.1	1378962611	1	176	1656	1831	26895939	26895764	AG<exon>GT	M176
+1	KF286658.1	1378962611	1	137	1832	1968	26894778	26894642	AG<exon>GT	M137
+1	KF286658.1	1378962611	0.987	151	1969	2119	26893393	26893243	AG<exon>GT	M21R2[illegible]
+1	KF286658.1	1378962611	1	169	2120	2288	26892646	26892478	AG<exon>GT	M169
+1	KF286658.1	1378962611	0.994	155	2289	2443	26892363	26892209	AG<exon>GT	MRM15[illegible]
+1	KF286658.1	1378962611	0.984	124	2444	2567	26891837	26891714	AG<exon>GT	M37RM[illegible]
+1	KF286658.1	1378962611	0.986	146	2568	2713	26890567	26890422	AG<exon>GT	M124R[illegible]
+1	KF286658.1	1378962611	1	131	2714	2844	26890296	26890166	AG<exon>GT	M131
+1	KF286658.1	1378962611	0.971	102	2845	2946	26889676	26889575	AG<exon>GT	M48R2[illegible]
+1	KF286658.1	1378962611	0.989	92	2947	3038	26887511	26887420	AG<exon>GT	M52RM[illegible]
+1	KF286658.1	1378962611	0.972	249	3039	3287	26886933	26886685	AG<exon>	M4R2M[illegible]

Figure S3. Tabular format for the alignment between Vechur ATP1A1 sequence KF 286658.1 and Bos taurus reference genome (1378962611).

```
>lcl|Seg1    gb|KF286658.1| [1,13] x gi|1378962611
TATGGGGAAGGGG
>lcl|Seg2    gb|KF286658.1| [14,118] x gi|1378962611
GTTGGACGTGATAAATATGAACCGGCAGCTGTTTCAGAGCATGGGGACAAGAAAAAAGCCAAGAAAGAGA
GGGATATGGACGAGCTGAAGAAAGAAGTTTCTATG
>lcl|Seg3    gb|KF286658.1| [119,178] x gi|1378962611
GATGATCATAAACTTAGCCTTGATGAACTTCATCGCAAATATGGAACAGACTTGAGCCGA
>lcl|Seg4    gb|KF286658.1| [179,382] x gi|1378962611
GGCTTAACAACTGCTCGAGCTGCCGAGATCCTAGCCAGAGATGGACCCAACGCCCTCACGCCCCCTCCCA
CGACCCCTGAATGGGTCAAGTTCTGTCGACAGCTGTTTGGGGGGTTCTCAATGTTACTGTGGATTGGAGC
AGTTCTTTGCTTCCTGGCCTATGGTATCCAAGCTGCTACAGAAGAGGAACCTCACAATGATAAT
>lcl|Seg5    gb|KF286658.1| [383,496] x gi|1378962611
CTGTACCTTGGTGTGGTGCTGTCTGCTGTCGTCATCATAACCGGGTGTTTCTCCTACTATCAAGAAGCTA
AAAGCTCAAAGATCATGGAGTCCTTCAAAAACATGGTTCCTCAG
>lcl|Seg6    gb|KF286658.1| [497,631] x gi|1378962611
CAAGCCCTCGTGATTCGAAATGGTGAGAAAATGAGCATAAATGCGGAAGAGGTTGTCGTCGGAGATCTGG
TGGAAGTGAAAGGAGGGGATCGAATTCCTGCCGATCTCAGGATCATTTCTGCAAATGGCTGCAAC
>lcl|Seg7    gb|KF286658.1| [632,749] x gi|1378962611
GTGGATAAGTCCTCACTCACGGGAGAATCAGAGCCACAGACCAGGTCTCCGGATTTCACCAATGAAAATC
CCCTGGAGACACGGAACATTGCCTTATTTTCAACTAAGTGTGTTGAAG
>lcl|Seg8    gb|KF286658.1| [750,1018] x gi|1378962611
GCACTGCACGTGGCATTGTCGTGTATACTGGGGATCGAACCGTGATGGGCAGAATTGCCACGCTGGCTTC
TGGGCTGGAGGGCCGCCAGACTCCCATCGCTGCCGAAATTGAACATTTTATCCACATCATCACGGGTGTG
GCGGTGTTCCTGGGCGTGTCGTTCTTCATCCTTTCTCTGATCCTTGAGTACACCTGGCTGGAGGCTGTCA
TCTTCCTCATCGGCATCATTGTAGCCAACGTGCCAGAGGGTTTGCTGGCCACCGTCACG
>lcl|Seg9    gb|KF286658.1| [1019,1217] x gi|1378962611
GTGTGCCTCACGCTGACTGCAAAGCGCATGGCCAGGAAAAACTGCTTAGTGAAGAAACTGGAGGCTGTGG
AGACCTTGGGATCCACGTCCACCATCTGCTCGGACAAAACTGGCCCTCTGACCGAGAACCGAATGACAGT
GGCCCACATGTGGTTCGACAACCAAATCCACGAGGCGGACACGACAGAGAATCAGAGCG
>lcl|Seg10    gb|KF286658.1| [1218,1327] x gi|1378962611
GTGTTTCATTTGACAAGACTTCGGCCACGTGGCTCGCTCTGTCCAGAATTGCAGGTCTTTGTAACAGGGG
CGTGTTTCAGGCTAACCAGGATAACCTGCCTATCCTGAAG
>lcl|Seg11    gb|KF286658.1| [1328,1462] x gi|1378962611
CGGGCTGTAGCGGGTGATGCCTCAGAGTCTGCGCTCCTGAAGTGCATTGAGGTGTGCTGCGGTTCTGTGA
AGGAGATGAGAGAGAGATACACCAAGATCGTCGAGATCCCATTCAACTCCACCAACAAGTACCAG
```

```
>lcl|Seg12   gb|KF286658.1| [1463,1655] x gi|1378962611
TTATCCATCCACAAGAACCCCAATGCAGGTGAGCCCCGCCACCTACTGGTGATGAAAGGTGCCCCAGAAA
GGATCTTGGACCGCTGCAGCTCCATCCTCATCCATGGCAAGGAGCAGCCCCTGGACGAGGAGCTGAAGGA
CGCTTTTCAGAATGCCTACCTGGAGCTGGGTGGCCTCGGAGAGCGTGTGCTGG
>lcl|Seg13   gb|KF286658.1| [1656,1831] x gi|1378962611
GTTTTTGCCACTTGCTGCTGCCAGATGAGCAGTTTCCTGAAGGCTTCCAGTTTGACACCGACGATGTGAA
CTTCCCTGTTGATAATCTCTGCTTCGTGGGACTCATCTCCATGATTGACCCACCACGGGCTGCTGTCCCA
GATGCTGTGGGCAAATGCCGAAGTGCTGGAATCAAG
>lcl|Seg14   gb|KF286658.1| [1832,1968] x gi|1378962611
GTCATCATGGTCACCGGAGACCATCCCATCACTGCCAAAGCCATTGCCAAAGGTGTGGGGATCATCTCAG
AAGGTAATGAGACTGTAGAAGACATTGCTGCCCGCCTCAACATCCCAGTGAGCCAGGTGAACCCCAG
>lcl|Seg15   gb|KF286658.1| [1969,2119] x gi|1378962611
GGATGCCCGGGCCTGCGTGGTGGATGGAAGTGATCTGAAGGACATGACTCCCGAGCAGCTGGATGACATC
TTGAAGTACCACACAGAGATTGTGTTCGCCAGGACCTCTCCGCAGCAGAAGCTCATCATTGTGGAAGGCT
GCCAGAGACAG
>lcl|Seg16   gb|KF286658.1| [2120,2288] x gi|1378962611
GGTGCCATCGTGGCTGTAACTGGAGACGGTGTCAATGACTCCCCGGCTTTGAAGAAGGCAGACATTGGGG
TTGCCATGGGGATTGCAGGCTCAGACGTGTCTAAGCAAGCTGCTGACATGATTCTCCTAGATGACAACTT
TGCCTCAATTGTGACTGGAGTAGAGGAAG
>lcl|Seg17   gb|KF286658.1| [2289,2443] x gi|1378962611
GACGTCTGATCTTTGATAACTTGAAGAAATCCATTGCTTATACCCTCACCAGTAACATTCCTGAGATTAC
CCCCTTCCTGATATTTATTATTGCAAACATTCCACTCCCTCTGGGGACCGTCACCATCCTCTGCATCGAC
TTGGGAACGGACATG
>lcl|Seg18   gb|KF286658.1| [2444,2567] x gi|1378962611
GTCCCTGCTATCTCCTTGGCTTATGAGCAGGCTGAGACCGACATCATGAAGAGGCAGCCCAGAAACCCCC
AGACGGACATACTCGTGAACGAGCGGCTGATCAGCATGGCCTATGGACAGATTG
>lcl|Seg19   gb|KF286658.1| [2568,2713] x gi|1378962611
GTATGATCCAGGCCCTGGGGGGCTTCTTCACTTACTTTGTTATCATGGCTGAGAACGGCTTCCTCCCAAA
TCACCTGCTGGGCATCCGAGTGACCTGGGATGACCGTTGGATCAACGATGTGGACGACACCTATGGGCAG
CAGTGG
>lcl|Seg20   gb|KF286658.1| [2714,2844] x gi|1378962611
ACCTATGAACAGAGGAAGATTGTGGAGTTCACCTGCCACACGGCCTTCTTTGTCAGTATCGTGGTGGTGC
AGTGGGCTGACCTGGTCATCTGCAAGACCAGGAGGAATTCTGTCTTCCAGCAGGGGATGAA
>lcl|Seg21   gb|KF286658.1| [2845,2946] x gi|1378962611
GAACAAGATCTTAATATTTGGCCTCTTTGAAGAGACAGCCCTTGCTGCAATCCTTTCCTACTGCCCTGGA
ATGGGTGTTGCCCTGAGGATGTATCCCCTCAA
```

```
>lcl|Seg22   gb|KF286658.1| [2947,3038] x gi|1378962611
ACCTACCTGGTGGTTCTGTGCCTTCCCCTACTCTCTTCTCATCTTCGTGTATCACGAAGTCCGGAAGCTC
ATCATCAGGCGACGTCCTGGCG
>lcl|Seg23   gb|KF286658.1| [3039,3287] x gi|1378962611
GTTGCCTGGAGAAGGAGACCTACTACTAGACCCCCTCCTGCACGCCGTGGAGCATCATGCCACAAACTCT
GCACCCACGACCCACCCCCTCTTTGTGTACTTCATTCTTGGAATTCAGAATTCCACGCTGGTAGGAAAGC
ACCGAAGCATGTGGGGAAGGGAGACGTCCCGGAATGAAGCATGTAGCTCTATGGGGGGGGTGGGGGTGGG
AAGAGGGGTGCCCGAGAAACATCCGTCTGTGGAAACGAC
```

Figure S4. Segments and boundaries of the aligned regions of 23 exons between Vechur ATP1A1 sequence (KF 286658.1) and Bos taurus reference genome (1378962611).

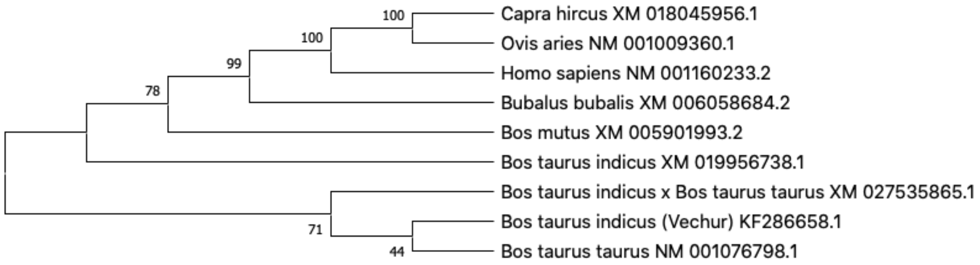

Figure S5. The evolutionary history of ATP1A1 gene in Vechur cattle inferred using the Neighbor-Joining method. The optimal tree with the sum of branch length = 0.12680380 is shown. The percentage of replicate trees in which the associated taxa clustered together in the bootstrap test (1000 replicates) are shown next to the branches.
